# Selections and Abstracts

**Published:** 1899-08

**Authors:** 


### SELECTIONS AND ABSTRACTS.

### EUROPEAN MEDICINE ABOUT 1799.*

In the first part of the eighteenth century general culture, the natural sciences and medicine underwent gradual but great changes; medicine, however, not always in proportion, for in England Locke's skepticism and Shaftesbury's and Bolingbroke's philosophies did not favor its development to the same degree as did Newton's mechanical foundation of physics, which trained the minds to look for the relations of cause and effect. The brilliant Jesuitism which controlled the mental condition of Italy benefited the upper classes only. That is why in that country natural

*Read by A. Jacobi, M.D., LL.D., New York, at the Centennial meeting of the Medical and Chirurgical Faculty of Maryland, held at Baltimore, April 25–28, 1899.

sciences and medicine had to be their own pathfinders. Holland was tolerant, but indifferent. The culture of France, then greatly under the influence of Locke, was an example and model to all Europe, but science and refinement belonged exclusively to the aristocratic circles. It was only the encyclopedia, the first volume of which appeared in 1751, that, while it afforded the intellectual basis of one of the most beneficent volcanic eruptions in popular evolution, the French Revolution, fostered medicine as it did all sciences. Germany, in the beginning of the eighteenth century, had not even a scientific language of its own. Latin was the organ of erudition and of teaching, and exhibited all the pedantry that could be squeezed into a dead language. One of Germany's great men, Leibnitz, wrote, when not in Latin, in French; only one, Wolf, either in Latin or in German.

The signature of those times was the incomplete knowledge of actual facts. Imperfect reasoning was its unavoidable result. While in our days psychology is becoming a part of physiology and physics, thinking at that period was metaphysical throughout.

Cartesius recognized one thing only that was actual—the power of thinking. "*Cogito ergo sum.*" Thinking is to him the source of every knowledge. Leibnitz elaborated this dogma contrary to the sense of Democritus. This ancient philosopher constructed the world on the foundation of matter; Leibnitz on that of ideal, indivisible atoms, which were its power-centers. Both Cartesius and Leibnitz, however, had in their nebulous tendencies a reconciling element in this, that they insisted upon mathematics and physics as the basis of study. Kant, though he urged the importance of experience—*Erfahrung*—concluded that whatever entered into our senses did not correspond with the actual facts, but furnished phenomena—*Erscheinungen*—only. Thus he and his followers, for instance Fichte, relied on intellect—*Vernunft*—as the foundation of science. The latter philosopher arrived at the final result that the external world was only the production of the intellect. Such deductions and imaginations were not without their unfavorable influence on medicine. One of the greatest physiologists of this century, Johannes Müller, even he taught that what we perceive or know are not realities, but only the impressions on the retina or other nerves. Thus it could happen that Schelling, imbued with the philosophical doctrines of two centuries and under the influence of the teachings of John Brown and of the systematic perversion of Haller's "sensibility" and "irritability," demanded that science should be based on and constructed out of the working of the intellect only. Facts were replaced by wanton hypotheses; pathological processes were explained by the not-understood action of the nervous system. What little was known of galvanism and magnetism was utilized in the interest of verbose assertions. "Polarity" between light and gravity, between head and the lower parts of the body, chest and abdomen, arteries and veins, nutrition and secretion, left and right half of the body, were high-sounding words used to cover ignorance and lack of system. It was this food that nourished German medicine in the first forty years of this century, with its crudities, absurdities and pomposities, with words instead of facts, with wild theories and unintelligible phrases, with wanton assertions in place of observations. The diagnosis of those times were asthenia and hypersthenia; exhaustion and perversion of vitality; the gastric, bilious, rheumatic, catarrhal or nervous state; rheumatico-catarrhal sub-gastric fever; gastrico-bilious subnervous fever; gastrico-nervous inflammatory fever. Therapeutics was under the influence of Brownianism and mostly exciting. Haeser relates: In 1798 Marcus, in the hospital of Bamberg, had 480 patients, of whom forty-six had sthenic, 367 asthenic and sixty-seven local disorders. The average medication of every patient was as follows: One drachm of opium, 195 grains of camphor, one ounce of liquor anodynus, 132 grains of serpentaria, 528 grains of cinchona bark, more than one pound of rectified spirits and quantities of musk, naphtha vitrioli, arnica, valeriana, angelica, cannamon, tincture martis tonica and elixir roborans Whyttii.

The same lack of intellectual preparation and solidity caused the many theories invented for the purpose of explaining the phenomena of life.

Glisson was the first to look for a common vitalistic influence as an explanation of organic forces; Hoffman sought for it in the nervous system, and called it ether; Stahl in his hypothetical

"anima," which was the independent and only principle of life; the followers of Haller in irritability and sensibility, and Cullen and Brown and Rasori in the excited condition mainly of the nervous system. In France it was Sauvages, Bordeu, Barthez and, finally, Pinel who sustained the principle of vitalism, and it was only under the influence of Morgagni, the founder of pathological anatomy as related to the morbid processes in the body, and under that of the slow development of histology, that the three latter began to reason from pathological points of view. It was Barthez who urged that a single vital force was not sufficient to explain all the phenomena of life in the different organs, and attempted a subdivision of the vital force into a plurality of forces, and Bordeu, who observed the structural difference in the composition of organs, thus paving the way for Bichat, one of the great pathfinders in medicine, at the dawn of the new century.

When Haller studied the functional differences in the organs, he found in the muscle, when excited, its capability of contracting, and called it irritability, a term invented by Glisson, with whom it means the reaction exhibited by the body under the influence of excitation.

This simple and great discovery was too much for the unprepared minds, who could not understand the imminent connection of force and matter. That is why irritability was supposed to be a thing of its own, an external power which moved the muscle. Nor was this all. The definition of irritability was extended to the rest of the organism; wherever there was action, or the energy of resistance, there was the alleged independent irritability behind it (not in it). As its contrast they established sensibility, that is, the passive exhibition of pain and unrest. These two terms of irritability and sensibility play an important part in the numerous writings which appeared as a mixture of Haller's irritability, Hoffman's nerve fluid and Cullen's neuro-pathology, and treated only of the solid parts of the body. Still, in the latter, it was not its structure and its functions that were considered and studied, but only the two supposed fundamental forces outside them, viz., irritability and sensibility. In this way inflammation and fever were diseases, not of the organism, but of irritability; typhoid fever was an exaltation of sensibility; septic fever the absence of irritability. With minds so obscured by words without contents, Hahnemann found it easy to introduce syphilis, psora and the merely hypothetical sycosis as the main sources of all diseases. Indeed, this postulate may have been welcomed by many as an escape from the presumed vital forces (which was looked upon sometimes as mere nerve fluid, other times as something superior and external to it, and which was thought to present both irritability and sensibility as the result of a dual "polarity").

It was then that Mephistopheles said in "Faust":

> "Just where fails the comprehension
> A word steps promptly in as deputy.
> With words 'tis excellent disputing;
> Systems to words 'tis easy suiting.
> On words 'tis excellent believing
> No word can ever lose a jot from thieving."*

The quintessence of the immense literature of that verbose time may be stated as follows: The structure of the body cannot explain its functions. The soul cannot explain them. That is why there must be a third, the vital force, which resides in the brain and the nerves. Alone Blumenbach ("Institutiones Physiologicæ," 1786) refused to limit the vital force to the nervous system. Every organ, according to him, with the exception of the blood, has its own life. Alongside irritability and sensibility, he established a plastic, or formative, or reproductive power (*nisus formativus*), and succeeded in reintroducing the neglected topics of nutrition and metabolism into the horizon of medical thought.

But not even he could stem the current of mostly German obtuse literature, which had no room for and no thought of exact observations. It took half a century to re-establish common sense, intelligible terminology and straight thinking into German medicine under the slow influence of French creative enthusiasm, first kindled by Broussais, and of British cool conservatism.

The infected mental atmosphere produced two peculiar systems, the modifications of which claim our attention this very day. They are Mesmerism and Hahnemannism. Mesmer was not always a fraud. His inaugural thesis of 1766 ("*de influxu planetarum in*

*Bayard Taylor's Translation of Faust.

*corpus humanum*") betrays a mind clouded by mysticism. In later times he treated diseases not only by the direct influence of animal magnetism, but also at a distance. There is a magnetic fluid in every organism; its existence is the link between different bodies, and thus both physical touch and spiritual influence cause relief from pain and convulsions and produce somnambulism and clairvoyance. In France it was only Paris that ran mad in spite of the adverse report of the Academy; in Germany mesmerism was at once taken up by the thousands of medical men who were infected with the philosophical theories then afloat. Then there was what was called "spiritual concubinage" and "spiritual generation," there was "polarity" between the magnetizer and the magnetized, between the "solar" brain and the "telluric" ganglionic life. Mesmerism always found its main apostles in Germany, like spiritualism, hypnotism, clairvoyance, Christain faith amongst the mentally inferior classes of modern America. My old ingenious teacher, Friedrich Nasse, in Bonn, while being amongst the foremost to introduce into Germany the exact methods of Laennec, and trying his and my hands on experimentation, still proposed in 1850 that I should go to Holland to treat with animal magnetism a young lady in whom he was interested.

Hahnemann began his career with a paper published in 1796. Within a few years he completed his teachings, the principal of which were as follows:

The only vocation of the physician is to heal; theoretical knowledge is of no use. In a case of sickness he should only know what is curable and the remedies. Of the disease he cannot know anything except the symptoms. There are internal changes, but it is impossible to learn what they are; symptoms alone are accessible; with their removal by remedies the disease is removed. Their effects can be studied in the healthy only. They act on the sick by causing a disease similar to that which is to be combated, and which dissolves itself into this similar affection. The full doses required to cause symptoms in the well are too large to be employed as remedies for the sick. The healing power of a drug grows in an inverse proportion to its substance. He says literally: "Only potencies are homeopathic medicines." "I recognize nobody as my follower but him who gives medicine in so small doses as to preclude the perception of anything medicinal in them by means either of the senses or of chemistry." "The pellets may be held near the young infant when asleep." "Gliding the hand over the patient will cure him, provided the manipulation be done with firm intention to render as much good with it as possible, for its power is in the benevolent will of the manipulator." Such is the homeopathy of Hahnemann, which is no longer recognized in what they call homeopathy to-day. The present apparent heresy is legitimate enough, like most gradual changes. Unsectarian science itself has changed its principles and doctrines until it arrived at the basis of strict observations and well-planned experimentation.

Hahnemann's learning and intelligence made him familiar with the onesidedness or incompetency of all the theories and systems which had controlled the medical market of the century. His new system announced with ferocity, and appearing unintelligible and crude to a sound mind, could not but impress the multitude which did not differentiate between one bad logic and the other. His contempt for actual observations and experience pleased the ignorant, his violent criticisms of everything preceding him appealed to the unschooled Protestant mind; indeed, he compared his cause with the fight of Protestantism against Catholicism; his very violence tallied with the revolutionary spirit of the time; the mysticism of the medicinal power of a substance when reduced to an unthinkable minimum was a stunning exemplification of the superiority of the spirit over the body; the ridicule and persecution to which his teaching and his vehemence exposed him were claimed as martyrdom.* Thus it happened that he gained not only a foothold, but founded what is called a school exactly 100 years ago, and secured an immortality for his own name and the title he invented for his doctrines, for that title, however, has outlived the practice of his teaching, which began to change and degenerate, and was objected to by those who in the course of generations learned to cling to nothing that was his, except the name.

Theories and systems are not by themselves necessarily harmful

*Tagel, "Geschichte der Medicin"

as long as they act merely as mental gymnastics. As soon, however, as they are applied to the explanation of actual facts, and claimed as the rules for therapeutical interference, they cripple observation and are misleading. A mere philosophical theory is like a dogma, not by itself injurious unless applied to realities. Natural facts, medical truths, economic laws are the same, whether applied to the adherents of Moses, Confucius, Christ or Mohammed. Even positive religions do not necessarily interfere with science, and science does not necessarily interfere with religions. How little there is to fear from dogma or theory when the mind is otherwise clear and guided by actual observation, is well instanced by Sydenham, one of the greatest physicians of all times. It was he who laid particular stress on diet, restrained the gruesome medieval medication, limited depletion, and introduced cooling methods of treatment. And here follows his reasoning. For the effect of his treatment he looked in the "smothering of animal spirits, which are the primary instruments of concoction."

Another instructive specimen of the amplification of vitalism with fairly sound judgment is exhibited by Cullen. According to him, who lived nearly to the end of the eighteenth century, the nervous system is the source of life and of all diseases. It is through the nervous system that remedies show their action. Mechanical and humoral forces are of very rare influence. Cullen accepted Hoffmann's division of morbid conditions into spasms and atony. In fever the chill is its prodrome and its cause; the chill is a debility of the brain; the peripherous ends of the blood-vessels are spasmodically contracted. In inflammation the essential symptom is congestion into the blood-vessels. Amongst the neuroses are the comatose diseases, adynamies, spasms, psychical diseases and topical disorders. Only in scrofula and scurvy is there an alteration of the fluids. His therapy is simple; the drugs act on the nerves, which are either stimulated or weakened.

His pupil, Gregory (†1822), without adding much to Cullen's teaching, controlled forever the simplicity and sobriety of English medical thought. While no country suffered more from theories than Germany, England was nearly immune.

Cullen's teaching was followed and sustained on the Continent mostly by de la Roche of Geneva and Paris (1743-1815), who was an out and out neuropathologist, and in part by Vacca Berlinghieri of Pisa. It was he who caused solido-pathology to take precedence in German pathological thought. Altogether, however, the English practitioners were not partial to theories; their own Brown they left to other countries to go mad over. Cheselden, Benj. Bell, Fothergill, Pringle, Heberden, Home were straightforward and cool observers. Currie, after the example of Hahn on the Continent, taught the use of water as a powerful therapeutic agent. Tissot in France, Zimmerman, Quarin and J. P. Frank in Germany, Austria and Italy, furnished good histories of diseases and diagnosis which considered the physical totality of the patient, not a single symptom only.

While the eighteenth century was one of theories and of symptoms, all of which were to pass away, there was also a large amount of practical work. The Academy of Surgery was founded in Paris by Marèchal, 1731, and endowed in 1741 with rights equal to those of the medical faculty. The "Practical School of Medicine" was established in 1738. When I mention the names of the first teachers, Chopart and Desault, the rapid development of surgery in France, and under its influence in Great Britain, becomes explainable. Germany was slow in following; its surgery was inferior to, though influenced by, that of France and England until the middle of the nineteenth century, in spite of Lorenz Heister, Schmucker, Theden and Richter; in spite of Haller, the great and broad man of the century, who taught anatomy, botany, chemistry, medicine and surgery, the latter, however, without the courage to operate; in spite of the foundation of the Charité by Frederick, of the Josephinum by Joseph of Austria, and in 1895 of the Medico-Surgical Frederick William Institution of Berlin.

In the second half of the century obstetrics also made a great stride. It is true that in 1746 the Medico-Pharmaceutical College of Amsterdam prohibited obstetrical practice unless the practitioner bought, at a cost of 2,000 florins or more, a secret instrument from the same college. When its nature finally became known it proved to be a simple lever. In 1747, however, Levret described the forceps Palfyn had presented to the Paris Academy in 1723. All that time it had remained unknown. De la Mott and Baudelocque are, beside Levret, the most prominent French, Manningham, Smellie, Wm. Hunter and Denham the most influential English obstetricians. The obstetrical literature was very large; nowhere more so than in Germany, where Mettelhauser could boast that while in every labor requiring aid the child was always lost—that was self-understood—he succeeded in saving two out of ten women. How many of the two died afterwards of puerperal fever we are not told. The cruel mortality was finally the reason why obstetrical schools were founded in many German towns. Undoubtedly the midwives thus instructed have contributed to the preservation of many lives. In our time, and in our country, while the profession's brightest honor consists in its labors in behalf of sanitation and prevention, the teaching of simple midwifery is tabooed by that very profession.

The second half of the century furnishes a large number of monographical publications. The mind, eclampsia, chorea, catalepsy, hysteria, neuralgia, apoplexy, the spinal cord, the heart, the lungs were often treated of. Senac on the heart, 1749, Auenbrugger's Inventum Novum of 1761 should always rank amongst the classics; so should Home on croup, 1765, Baumès on Tabes Mesenterica, 1788, Wichman on the "Etiology of Itch," who in 1786 drew the acarus, Cardogen, 1771, and Grant, 1781, on gout, Huxham, Pringle, Sarcone and Campbell on typhus, Huxham on "Febris Nervosa Lenta," and Roederer and Wagler De Morbo Mucoso, 1762, both of which were evidently meant for typhoid fever. There are many more whose names a grateful history should not forget, too many to be mentioned in this sketch.

But no sketch ever so brief can miss the name of Edward Jenner. It was on the 14th day of May, 1796, that he vaccinated a boy named James Phipps with pus taken from Sarah Nelms, a dairy maid who suffered from cowpox, and in 1798 he published "an inquiry into the causes and effects of the variola vaccina, a disease discovered in some of the western counties of England, particularly in Gloucestershire, and known by the name of cowpox."

The most fertile progress in medical thinking was, however, made through new researches in pathological anatomy and histology.

Morgagni (1682-1771), *de sedibus et causis morborum*, 1761, connected with the results of autopsies with the processes observed during life. In the words of Virchow, he introduced anatomical thinking into medicine. It was he that facilitated the labors of John Hunter, who appreciated the significance of pathological anatomy and of experimental research, and established the museum which alone would suffice to carry his name to all future generations. Nor is it probable that without Morgagni, Bichat could have evolved his discoveries, he the solitary histologist without predecessors—Pinel and Bordeu perhaps excepted—and for many decades, even in his own country, without successors, just 100 years ago. His new doctrine coincides with the dawn of a new century. He taught that every tissue differed from the rest in its vital properties; that is why its diseases also should differ from one another. That is also why an organ composed of several tissues might exhibit a disease in one of its tissues only, while the rest remained intact or nearly so. Still in so far as the contact and connection between the components of an organ was intimate, a morbid condition could migrate from one to its close neighbor; for all these reasons pathological anatomy must begin with the discussion of the tissues.

The eighteenth century, with means more limited than ours, with imperfect or no instruments of precision, has accomplished much for medicine; partly conjointly with increasing general culture, partly through the exertion of a few superior men. In this respect science differs from the rules governing the political evolution of the human race. In science history can be made by a single brain; in politics but rarely. Alexander succeeded in part. Not even Julius Cæsar accomplished it. Napoleon, though powerful enough to shake the universe, fiendish enough to murder defenceless prisoners of war, and reckless enough to depopulate his own country, could not do better than extend the lessons and results of the revolution which he aided in throttling. Bismarck, though far-seeing, unscrupulous, inconsistent and brutal, accomplished but a part of what the bleeding and starving youth of 1848 had prepared for him. It is only our country that is so fortunate as to possess a few men so great that it seems it could not have thriven without them. Washington was of that type; also that fearless, sturdy and uncompromising statesman, the involuntary choice of a reluctant party, whose dignified retirement speaks as loud for his greatness as his vigorous steering when he controlled the helm, unmoved by the waves.

In science single men do make history. So did Paracelus when he shook off the yoke of Galen, who had reigned 1,600 years. So did, towards the end of the eighteenth century, just previous to the foundation of your faculty, Morgagni, when he introduced anatomical thinking into medicine; Haller, when he taught the functions of different organs, mainly the muscles, and discovered the existence of sensitive nerves, and of nerve currents going in different directions; John Hunter, when he established pathological anatomy and experimentation on a sound basis; Jenner, when he laid the foundation of sero- and organo-therapy; Bichat, when he created histology. That is what Virchow did when he fixed the throne of life in the invisible cell; Pasteur when he demonstrated forever the omnipresence and omnipotence of the unseen microbe. "Narrow is the universe," so the poet says, "when compared with the vastness of man's brain."

The social position of the physicians became much improved during the eighteenth century. In its beginning it was decidedly low. The public at large was not in contact with the few illustrious men who had the monopoly of positive knowledge and of sound judgment, but with the crowd of ignorant, quackish or superstitious practitioners. About 1750 circumstances improved very much, and towards the close of the century the medical profession enjoyed the confidence and veneration of the public to a high degree. Riches they had not, for it is true there were but few physicians who became opulent; still a small income sufficed for a competency. The veneration and confidence, and the influence in all matters, terrestrial and celestial, formerly possessed by the clergy, were now enjoyed by the doctor. The growing indifference in matters of positive religion relegated the clergy to the pulpit, to the death-bed and sacred ceremonies, and the absence of the confessional from protestant countries made of the family physician the father confessor, the adviser and friend in matters physical, mental and emotional. Gradually he also became the adviser of the commonwealth, for the influence of medicine became more tangible in the interests of public health when forensic medicine and medical sanitation were first written about amongst other celebrities by J. P. Frank. Thus obedience to the physician became a matter of course, and the appreciation of his superiority self-understood. Even eccentricities in manners and dress and charlatanism, which fostered the belief in his occult knowledge and secret arts, did not hurt him in the estimation of the many, while it is true that through them the better-informed and better-mannered portion of the public became doubters or adversaries. These were sustained in their scorn by what was going on in the profession itself. It is true that only the best informed could appreciate the contradictions between medical theories, and that what Stoll called salvation, C. L. Hofmann called perdition ; that Brown cursed them all, or that medical books could not be printed in Germany without undergoing the censorship of a university like a Philippino war despatch. But there were things that the plainest mind could grasp. One of the greatest public scandals of the century was the steal of Girtanner's, who succeeded through years to pawn off John Brown's teaching as his own. Then came Hahnemann. He and his immediate followers had a more injurious effect on the relations of the medical profession to the public in this, that they did not present a new doctrine for its own sake, a new system which was to enlighten the world, but a creed like Mohammed's in behalf of which the world, the medical world, was to be fought. The talk of the demagogue and the vile language of the street were his weapons. Books and pamphlets were written in which the laymen were called upon to pass judgment. The attacked party was careless enough to do the same. In this way the laymen were by both parties, made to believe that neither learning nor experience was required for a complete understanding; that, on the contrary, they would bias a correct criticism. Since that time more than ever before, the public knows it all, while the medical man learns by hard work that he knows but little. Since that time the newspaper reporter is the medical authority, and the neighboring lady friend the consultant. A few years ago a great medical editor proclaimed in a daily paper that the young newspaper reporters were the most appreciative and knowing critics of the tubercle bacilli demonstrations.*

The worst enemies, however, of the European profession a hundred years ago were its own members. Their manners were not above those of their fellow citizens, or as the case might be in different countries, their fellow subjects. Culture both of mind and heart was a monopoly of a few; the economic and mental distance between the aristocracy and the plebeian was immense. There was no dissemination of at least a minimum of instruction amongst the millions like at present. It is true that teaching the brain need not and does not improve the heart; a ruffian born will never be a gentleman, though ever so thoroughly drilled. The continental medical man of the last century spoke Latin, but he was for all that a man of his century. He was no longer Molière's polite scoundrel who in the consulting room said to his colleague: "If you let me purge him I shall let you bleed him." He appears, from all we know, and from what we conclude from the copious literature of the subject, rather coarse, self-willed, captious, jealous and noisy. The behavior in consultations between doctors was discussed in books and essays; that very fact proves that it required criticism and correction. I think we in 1899 have reason for self-congratulation when an author who wrote in 1791 declares consultations between doctors to be impossible, purposeless, time-killing, revolting and "lacerating." And in 1783, over his own name (Scherf's Archiv.), famous J. P. Frank advises in all seriousness the calling in of the police to arbitrate and restore order when doctors disagree in their consultations.

That explains why the literature on the relations of physicians to each other was very large in every country. Whatever is settled or self-evident is not written about. When good behavior or morals is universal there is no need of preaching or thundering

*Of C. A. Wunderlich, Geschichte der Medicin, 1859.

against the contrary. L. Stieglitz of Germany wrote (1798) appealingly and in a gentlemanly strain on the meeting of physicians at the sick-bed and on their mutual relations in general. Percival of England was more practical, inasmuch as he ordained in 1807 a code of ethics which was made the law book of the American Medical Association in 1847. Such a book should be taken as the expression of the best moral instincts and practices of the men superior in mind and heart. It is only after decades or generations, when general culture increases, that men will find their doings gradually approaching or reaching what the intellectual and moral nobility of their profession before them deemed proper and dignifying.

In gentlemanliness, candor, equity, helpfulness, consistency and principle, good men of all times and all professions are unanimous. No climate, creed, philosophical school, political party make any difference. Hippocrates, Aristotle, Plato, Aristides, Kimon, Æmilius Paulus, St. Paul, Lessing, Lincoln followed the same laws, unwritten except in their human hearts and read by their own starlight not visible to the crowd.

To what extent a code diminishes temptation or transgression it is difficult to say. There are no statistical researches to show that those who believe that a gentleman does not require one, and that no law book ever made an honest man out of a degenerate or a criminal, commit more sins than their neighbors.

Looking backwards 100 years we find many reasons for admiration. In all times the main scientific progress was made by individual men, and in a lesser degree only by the army of inferior workers preceding or succeeding them. "When the kings are building the truckmen are kept busy."* In the eighteenth century physical and mental communication was difficult and slow. There were no telegraphs and no railroads to bring people together in international congresses or through the "associated press" of science, but there were plenty of jealousies, censorship of books and lack of scientific apparatuses. There was no biological method of study, but there were outsiders with philosophical theories and insiders with vitalistic doctrines. So much the more do we admire

*Schiller, the colossal intellects and powerful workers, some of whose names I mentioned, that contributed so much to what we now are and have, and feel in duty bound to transmit to our successors for their eternal work. We have too many advantages in this work not to appreciate them. The greatest I know of is the dissemination of modern methods of research amongst all the members of the profession. In that way thousands are made to co-operate, where a hundred years ago there were a few. It is barely possible that the future will not produce many Bichats or Hunters or Virchows; aye, they may not be required, for those found the paths for us and secured our methods for all times. Besides, where the average size of men is great, the Sauls who rise above their shoulders become less in number. It may be quite possible, therefore, that of those who ornament the medical profession of this and other countries at the present time, very few or none will in a hundred or two hundred years shine through the dim past—simply because so many of the file have risen to rank. The greater the accomplishment of the masses the less is the opportunity of individual stars to outshine the pervading general light. What in former centuries gigantic men accomplished will be performed, I think, by great institutions. Not many individual names may be remembered, but the achievements of Harvard, Columbia, Ann Arbor, University of Pennsylvania, Johns Hopkins and the rest will be enumerated amongst the glories of the coming centuries. Single men may not be the predominant powers, but great societies like yours should and will take their places in history. The totality of great institutions and societies is like nature, always active, always effective, immortal. It is a wonderful mental apparition, this co-operation of a hundred thousand for a common end, that end being science and humanity. That is the way in which science will be the example for our political and social future. Like science, society furnishes the history of the association of individuals into a community, of these into a State, of States into the Union. Science, however, is ahead of politics. We do not speak any more of American or German or French science or medicine. They have become cosmopolitan. The illustrous names of England, France, Germany, Italy, are our names, as ours are theirs. Scurrilous allusions to nationality and yellow country jealousy, which it is true no longer linger amongst statesmen, but are characteristic of low politicians, have no room amongst us. Equality, fraternity and solidarity are inscribed on our flag. We recognize that the work of mankind is not performed by one man, by one country. The great principle for which this union was hammered out of jealousies and strife is also that of science as now understood, viz., enlightened, progressive and co-operative democracy.—*Maryland Medical Journal.*

---

### TREATMENT OF FRACTURES OF THE PATELLA.*

My experience with fractured patellæ has been limited to six cases, all of which except one, were treated by the operative method. Four of them were treated by open arthrotomy; one by Barker's subcutaneous suture.

I shall not attempt an exhaustive study of the comparative advantages of the various methods of treatment advocated by different writers. The literature covers a wide field, and much of it, like that on other surgical subjects, has become antiquated as modern ideas and methods prevail. To illustrate: Wythe, in his work on surgery, edition of 1887, says: "Many innovations have been made in the treatment of these fractures, some of which are unnecessary and unjustifiable." Aspiration is mentioned as one of the unnecessary innovations. He says: "The most unjustifiable method of treatment ever introduced in this fracture is that of opening the joint, and wiring the fragments together." It is only the more recent literature that is worth reading, and, I am sorry to say, there is much in this that will not bear the test of modern scientific observation. The text-books cling to some of these antiquated ideas so tenaciously that the reader may be misled as to their real worth. Recognizing the possible dangers in open arthrotomy, authors of text-books hesitate to give preference to this method of treatment. In the hands of those who are not competent, or those who have not the advantages of skilled assistants, and aseptic surroundings, there is, perhaps, a great deal of

*Read by Will J. Means, A.M., M.D., Columbus, O., before the Ohio State Medical Society, Springfield, May 10, 11 and 12, 1899.

danger both to life, and of permanent injury to the joint. While this conservative position is a commendable feature, it is strange that such instruments of torture as the Malgaigne hooks are given prominent notice.

The treatment naturally divides itself into the non-operative and the operative. Long experience has shown that fairly good results can be had from the non-operative treatment. It again shows that bony union of the fragments is not obtainable, that fibrous union is not permanent, and the functional activity of the patella is limited. Some authorities claim that the failures are due to faulty methods; but when we consider that the methods are almost as numerous as the writers, it is doubtful whether this assertion is literally true.

In the first place, it is practically impossible to get apposition of the fragments, and maintain it. The great extensor muscle of the leg that holds the upper fragment firm in its grip, is hard to extend and to immobilize. Again, the space between the fragments is filled with effused blood, synovia, and loose tissues. When the joint is opened, and the nature and extent of the débris filling up the space are seen, the surgeon can comprehend the difficulties in the way of apposition. The upper fragment often carries with it shreds of the aponeurotic tissues that drop into the space, and cannot be removed other than by mechanical means.

Another difficulty arises in fixing the fragments. The upper one especially, lies loosely beneath the integument, and cannot be held fast with any appliance over the integument without using a pressure that is not safe to the tissues.

In comminuted fractures the difficulties are much greater, and, I might say, insurmountable.

In one case of ancient fracture coming under my notice, and reported in this paper, the effused blood had become organized, and the joint very much hypertrophied therefrom. The lacerated capsule may also become attached to the surrounding fascia in such a manner as to destroy the normal function of the joint.

Again, the non-operative treatment is productive of a great deal of physical inconvenience, owing to the fact that the appliances to hold the fragments in apposition must of necessity produce considerable pressure, thus causing pain. The duration of the treatment is another inconvenience that must not be overlooked.

It is suggested by some authors that the effused blood be removed by aspiration, but when this is resorted to, the danger of infection is almost as great as open arthrotomy.

In justice to the advocacy of the non-operative treatment, it must be said that the knee joint is more prone to infection than the abdominal cavity; and it is only by the most strict aseptic technique that infection can be avoided.

Whatever form of splint is used, the fact must be taken into consideration that the disability is largely due to the contraction of the quadriceps muscle, and that this contraction must be overcome. The contraction of the ligamentum patellæ is limited. A splint made out of straw board is perhaps the simplest of construction and application, and yet as efficient as any that can be applied. A piece of straw board long enough to reach from the gluteal fold to the foot, and in width equal to one-half the diameter of the leg, is moistened on one side, and molded into a trough. Sufficient non-absorbent cotton to protect the integument from chafing should be used. At the joint a heavier pad should be made to relieve the leg from over extension, a very painful position. Before placing the splint, an elastic bandage should be applied over the joint. This will, in two or three days, reduce the swelling, and place the joint in condition for the application of fixation straps. These can be applied in various ways. The best, however, in my judgment is the rubber adhesive plaster, the upper piece cut in the shape of a U, and the lower one in the shape of a T. These pieces are applied with their expanded margins at the upper and lower borders of the respective fragments. A snug bandage should be applied to hold them in place. The fragments are then approximated, and the ends of the adhesive straps fastened; these again should be held in place by bandages applied in such a manner as to assist in holding the fragments together. The posterior splint should then be placed, and a firm bandage applied from the toes to the groin. I have found straw board to be economical and efficient for almost all kinds of splints.

There are other methods of treatment that perhaps deserve mention, but I have had no experience with them, and therefore do not care to discuss their particular merits. I might mention, however, inasmuch as the method should be used more or less in all cases, that the treatment by massage is now being advocated by some well-known surgeons. It is called the Dutch method, and was introduced by Titanus of Amsterdam in 1885. This method removes the objection of long confinement, and its advocates claim excellent results. However, there is no evidence presented that bony union takes place in cases thus treated. It claims that the function of the knee is more perfect than by any other non-operative method. For a description of this method I will refer the reader to an article by Charles A. Powers, of Denver, Colorado, published in the "Transactions of the American Surgical Association." It might not be out of place to say that this article is, perhaps, the most exhaustive one on the subject of fractures of the patella that I have found in medical literature.

My limited experience with the operative treatment has yielded such excellent results that I am ready to place myself on record as favoring it. I have seen a few cases treated by the open method that went wrong; but I was satisfied that the technique of the surgeon was faulty.

The technique of the operation is of great importance. It seems to me, however, that the one consideration that is paramount to all others, is that of asepsis. I shall not enter into any detail as to the preparation of the field of operation; this is well known to every surgeon. The incision opening the joint is perhaps a matter of choice. I have used the transverse incision and the longitudinal, and the results were practically the same. However, my choice is the longitudinal incision; I believe it to be the more scientific. The incision should be made of sufficient length so that when the edges are retracted the fragments may be fully exposed. This may require an incision four or five inches long; the length has no bearing upon the result. When the fragments are exposed the clotted blood should be thoroughly cleaned out, the shreds of lacerated tissue trimmed off, and the hemorrhage controlled by pressure with gauze sponges wrung out of hot water (no other material should be used for this purpose). Continuous irrigation with a hot saline solution should be used. Whether this acts as a preventive to infection, I am not prepared to state. It is my practice to make all arthrotomies under continuous irrigation of a salt solution. The uniform success obtained has led me to look upon it as an important part of the technique. After the lacerated aponeurosis and all loose tissues have been neatly trimmed, the fragments are drilled in three places. Three sutures properly placed are sufficient to hold the fragments in apposition. The drill should be started a quarter of an inch back of the margin, and come out anterior to the posterior surface of the patella. The suture material should be absorbable, therefore kangaroo tendon or catgut is used. The one difficulty that I have experienced with kangaroo tendon is its tendency to break in tying the knot. Silver wire should never be used. I have used it in many cases of fractured bones and have always had more or less trouble with it. After the sutures have been placed in position, the fragments are brought together, and the sutures tied. Great care should be observed that the joint is free from oozing. The aponeurotic tissues are then sutured with No. 1 catgut. I regard this as an important step. The sutured tissues add much firmness to the fractured patella. If left alone adhesions form, and the gap between the surfaces fills in with fibrous tissue, which materially affects the normal function of the joint.

The following is a report of the cases that I have treated:

Case 1.—Mr. S., aged 50, dissipated. Slipped and fell on the sidewalk. I saw the patient within two hours after the fall. Examination showed a transverse fracture of the patella of the right knee. There was no contusion of the skin. The fracture was evidently caused by the contraction of the quadriceps muscle. The fragments were separated about 1½ inches. The knee was badly swollen. The joint was aspirated and about eight ounces of blood removed. A subcutaneous suture of silkworm gut was passed around the fragments according to Barker's method. The fragments were brought together, and the ends of the suture tied externally. An elastic bandage was applied over the joint, and a posterior splint of straw board adjusted. After forty-eight hours the elastic bandage was removed. There was very little swelling. The fragments were practically in apposition. The suture was reinforced by adhesive plaster straps over the integument, and a plaster-of-paris splint was applied. The splint was removed in three weeks, and the suture taken out. A posterior splint was applied for several weeks afterward. The patient became insane and was taken to the State Hospital. I am unable to give the result.

Case 2.—Mr. T., aged 28, good habits. Fell from a ladder and struck on his left knee, producing a compound, comminuted fracture of the patella. Patient was taken home and treated there. The wound was carefully cleaned with tincture of green soap and boiled water. The edges of the lacerated tissues were carefully trimmed. All loose shreds of whatever kind were removed. The fragments were brought together with kangaroo tendon; five sutures in all were used. The field of operation was continuously irrigated with a hot salt solution during the manipulation. The edges of the lacerated capsule were united with fine catgut sutures. The integument was united with silk. No drainage was used. The joint was immobilized with a posterior perforated zinc splint. The joint was kept moist for several days with a solution of boric acid, alcohol and glycerine. On the seventh day the sutures were removed, and a dry dressing applied. The leg was then immobilized, and was not touched for three weeks. The splint was then taken off, and passive motion made. In three months the patient was using his leg, and able to flex it to an angle of 45 degrees. It was quite a year before he had normal mobility of the knee.

Case 3.—Mr. S., aged 32, good habits. While at work in the woods fell and broke the patella of left knee. Fracture was transverse. From the history of the case there was no direct force, and the fracture was evidently caused by a contraction of the extensor quadriceps. The fracture was treated according to the non-operative plan. After several months the dressings were removed. He went to work and while lifting a heavy load the fragments separated. He was then referred to me by his physician. I found a transverse fracture. Fragments 1½ inches apart. I made a transverse incision over the space between the fragments, The joint was filled with clotted blood. The fibrous band uniting the fragments had separated in the middle. The tissues around the patella were very much hypertrophied, due to the organization of the blood clots in the primary fracture. The joint was washed out, and the ragged edges of the aponeurotic tissues trimmed. The fibrous tissue covering the ends of the fragments was removed, and the bone exposed. The fragments were united then with kangaroo tendon, three sutures being used. The edges of the lacerated aponeurosis were united with fine catgut. The integument was closed with silk. No drainage used. Continuous irrigation with a saline solution was used during the operation. A firm bandage was applied to the joint, and a posterior splint of straw board immobilized the limb. The sutures were removed in eight days. The long splint was removed in four weeks. Passive motion with massage was used. The patient was alllowed to walk with a cane in three weeks from date of operation, and was able to use the limb in six weeks. In four months the knee was restored to its normal function.

Case 4.—Mr. L., aged 18, good habits. Fell on his knee producing a comminuted fracture of the patella. The integument was contused but there were no lacerations.

The joint was opened with a longitudinal incision. The fragments were sutured with catgut. The case was otherwise treated as cases 2 and 3. There was primary union and a complete restoration of the joint in three months.

Case 5.—Mr. L., aged 25, good habits. Fell from a tree. Left leg flexed under him. Patella broken transversely. I opened the joint with a longitudinal incision. The technique was the same as in cases 2, 3 and 4, except catgut was used for suturing the patella. A perfect result was obtained.

Case 6.—Mr. J., aged 50, laborer. Right patella broken by a fall from a wagon. Fracture transverse. Was treated by nonoperative method as described in this paper. Result poor. Function of the limb not good. Space between fragments apparently about one inch.

A record of six cases does not contemplate all the phases of fractures of the patella, but it does furnish sufficient data to justify me in claiming that the open method of treatment is a safe and efficient procedure.

Two of these cases were treated without operation primarily, and in both the result was bad. One required a subsequent arthrotomy; the other one, while the patient is going around, yet he has very indifferent use of his leg.

One case was treated with a subcutaneous suture, and when the patient was last seen, there was good evdence of a bony union of the fragments.

Absorbable material was used for all the buried sutures. The greatest care was taken in uniting the torn capsule. This I regard as one of the important steps in the technique.

Stimson, in his new work, says that the "Non-operative methods furnish in the great majority of cases in which they are properly used a result which is functionally satisfactory even if the union of the fragments is not close." With reference to the open treatment he says: "If there was no risk, it would deserve selection in almost every case." The American Text-Book of Surgery describes a number of operative measures, but gives no preference. DaCosta rather holds to the non-operative treatment, but mentions several methods of operating, placing special stress upon Barker's method. Dennis, in his "System of Surgery," speaks of the operative treatment, but points out the dangers that may arise therefrom, and describes at length the non-operative treatment. Wharton and Curtis, in their book, say: "The results obtained by the more conservative methods of treatment are usually satisfactory, and it is to be remembered that there is a definite amount of risk in exposing the patella and opening the joint," and further that no one is justified in suturing a fractured patella unless he is in a position to do an operation in which every aseptic detail is carried out. These authors all give great prominence to Malgaigne's hooks, and the various splints that have been used during the last half century.

CONCLUSIONS.

First. The results of the non-operative treatment are unsatisfactory both as to long confinement and functional disability.

Second. The methods of maintaining apposition of the fragments by external appliances are unsatisfactory and unscientific.

Third. In open arthrotomy the fragments can be carefully approximated and sutured in such manner as will maintain apposition and, ultimately, bony union.

Fourth. The operative method saves months of confinement, and gives permanent results.

Fifth. The buried suture material should be absorbable, such as catgut or kangaroo tendon.

Sixth. The field of operation, should be continuously irrigated with a hot salt solution during the manipulation, and the incision closed without drainage.

Seventh. The massage treatment begun at an early date is an important factor in restoring the functional activity of the joint.—*Columbus Medical Journal.*

---

### THE CAUSES OF DEATH OF PROMINENT PERSONS.

Dr. Ralph S. Michel made a collection of facts in regard to the deaths of celebrated persons. It was impossible, he says, to ascertain the cause of death in many instances, for frequently it was not known or stated; in many cases it was given simply as a fever. The following he believes to be quite accurate :

Early in the spring of 1616 Shakespeare and his boon companions, Ben Jonson and Michael Drayton, spent the evening at a tavern at New Place. All became too much intoxicated to reach home, and they remained out all night on the ground. The consequence to Shakespeare was a fever of which he died in a few days. It was undoubtedly pneumonia, Dr. Michel thinks.

Lord Bacon died at the age of sixty-five, a martyr to science. While out riding one winter day, it occurred to him that snow would preserve the flesh as well as salt. Accordingly he alighted, bought a hen, and stuffed it with snow. While doing this he became very much chilled and was too sick to return home. He stopped at a friend's house, where he was put in a cold, damp room, and he died in a few days, probably of pneumonia.

Burton, the author of "The Anatomy of Melancholy," believed in astrology. He calculated the time of his death by the stars, and he died at the time assigned, but he was suspected of taking something to hasten it in order to make it conform to his calculation.

Ben Jonson had several attracks of apoplexy. As a consequence his mental faculties became much impaired, and his last days were dark and gloomy.

Motherwell died at the age of thirty-eight of apoplexy.

Benjamin Franklin had gout, also cystic calculus, and the attendant inflammation of the bladder confined him to bed for a year. He was then eighty-four years of age. The immediate cause of his death was abscess of the lungs.

Washington, at sixty-seven years of age, died of acute laryngitis complicated with edema of the glottis. On December 12, 1799, he rode over his estate on horseback, and, as it was a day of rain and sleet, he became thoroughly chilled. He contracted a severe cold, and at the end of two days was very sick. Before sending for a doctor he had his overseer bleed him. When the doctor came he bled him again. As there was no improvement, a consulting physician was called in, and again he was bled. Finally they gave him tartar emetic and calomel; they also applied fly blisters to his throat. This medical treatment has been the subject of much criticism, Dr. Michel remarks.

Edward Gibbon, the historian, had the largest hydrocele on record—as large as a bucket. Repeated operations for relief exhausted him, and he died of a fever brought on thereby, at the age of fifty-seven.

Napoleon died of cancer of the stomach.

Thomas Grey, author of "An Elegy Written in a Country Churchyard," died at the age of fifty-seven. He was subject to hereditary gout. One day, at dinner, he was taken suddenly and violently sick with pain in the stomach, and died on the sixth day.

William Collins, the poet, died at thirty-six years of age, of paresis or insanity, brought on by dissolute and intemperate habits.

Akenside died suddenly at forty-nine years of age, of diphtheria.

Burns died at the age of thirty-seven. Of convivial habits, he perished from drink and exposure. One day in January, 1796, he died at a tavern in Dumfries. He was barely convalescent from a spell of sickness, and was in no condition to stand exposure. The night was cold, and Burns, wandering homeward in an intoxicated condition, sat down upon a doorstep and fell asleep. Rheumatism supervened, and, although he lived until the next July, he never recovered. During the last few days of his life he was in a state of low muttering delirium.

Byron was born with clubfoot. His mother, who was a misanthrope, always spoke of him as a lame brat. This defect was finally remedied to the extent of enabling him to wear a common boot. He early showed signs of obesity. This was to him a matter of much chagrin, and he combated the tendency by very low diet and medicine. He died in Greece, at the age of thirty-six, of heart complication, which set in during an attack of acute inflammatory rheumatism. Death was sudden.

Oliver Goldsmith died at forty-five years of age. He was a man of somewhat irregular habits, and was attacked with dysuria, probably amounting to strangury. His death was attributed to James' powders, which he would take contrary to the advice of his physician. But, as he died of strong convulsions, it is likely death was caused by uremia. James' powder was a secret remedy. It cannot now be prepared as it was by Dr. James, says Dr. Michel, owing to the ambiguity in the specifications placed on file in the Court of Chancery. But it is composed of antimonious acid and phosphate of calcium, with some sesquioxide of antimony. Dr. James was contemporary with Goldsmith, and the powder he used was probably prepared by Dr. James himself. If Goldsmith had perished from an overdose of it his symptoms would have been vomiting, purging and collapse.

James Beattie died of palsy.

Robert Fergusson, who was intemperate, died insane.

Chatterton died at the age of seventeen, of arsenical poisoning —a suicide. He was driven to the act by poverty and neglect.

Falconer, the author of "Shipwreck," was drowned by the foundering of a ship off the east coast of Africa.

Martin Luther died of violent inflammation in his stomach.

Cromwell died of remittent fever.

John Leyden died of the same fever, in Java.

Sir Walter Scott had several strokes of apoplexy. His memory failed, and softening supervened. The end came at sixty-one years of age.

Shelly was drowned by the capsizing of a boat in the Bay of Spezia.

Keats and Charles Wolfe both died of consumption.

Voltaire died of strangury, probably due to enlarged prostate. Very much has been said, says Dr. Michel, by ecclesiastics about the agony of his last days, as though it was a judgment for his outspoken agnosticism. In the days of 1778, when this condition received no treatment worthy of the name, what physician would doubt that the last days of Voltaire, who died when he was eighty-four years old, of strangury, must of necessity have been agonizing.

Galileo had stone in the bladder. With care, says Dr. Michel, he might have lived to shed upon that benighted time the rays of his intellectuality much longer. But he and the Church differed on astronomy. Galileo asserted that the earth traveled around the sun. The Church would brook no such heresy. Galileo was dragged out in the winter, jolted over rough roads in bad weather, to appear before the inquisition. Exposure, imprisonment and ill-usage killed him—a martyr to progress.

John Milton died at sixty-five years of age, of gout fever, or gout struck in, as it was called—our retrocedent gout.

Sir Issac Newton was long a sufferer from gout and stone in the bladder. He is supposed to have died from the latter.

Dean Swift once pointed to a dying tree and said: "I shall be like that tree—I shall die at the top." He had Ménière's disease, which produced paralysis, then aphasia, and finally a decay of all the mental faculties. He lived a year without speaking a word.

Edgar A. Poe was picked up off the streets of Baltimore one morning in 1840, and taken to a hospital, where he died without regaining consciousness; his death was attributed to drink and exposure. There has always been a suspicion that he may have been the victim of an assault. He was thirty-eight years old at the time of his death.

Lettia E. London—L. E. L.—died of an overdose of prussic acid.

Washington Irving died at seventy-six years of age, suddenly, of disease of the heart, in his own Sleepy Hollow.—*Indian Medical Lancet.*

---

### SYPHILIS—HOW SHALL WE STOP ITS ALARMING SPREAD?

Syphilis, one of the oldest and one of the greatest curses of mankind, has steadily gathered new victims, and now at this late day, the great syphilographers of the world are to meet at Brussels, Belgium, to devise some means by which to check its alarming spread.

The history of syphilis is a most appalling acknowledgment of gross disregard of its serious nature, and an unpardonable delay in a systematic and continual warfare against its propagation. It shows that the disease is most extensively met with in large cities in the very heart of civilization, and has on its muster-roll not only the prostitute and the habitué of the slums, but the aristocrat and those of the higher intellectual sphere; that it does not visit its wrath exclusively upon those who wilfully subject themselves to the danger, but also upon the innocent, for its mode of infection is not limited to direct venereal contact, but on the contrary, all objects which come in contact with the secretions of the mouth of a syphilitic may be the means of its transference from one person to many others, and that even infants may, through the medium of the nurse, fall victims to this dread disease. Notwithstanding this awful history, whose pages increase in numbers with each succeeding year, the world has seen fit to sit practically unconcerned and watch the disease spread, and the medical profession, with the exception of the syphilographers, has taken but scant interest in efforts to check it.

Effort to check the spread of syphilis is more the duty of the law than medicine. The physician has learned its origin, its nature and its treatment; he, from his position, can give no plan other than offer suggestions which must be accepted and acted upon by our legislative bodies. So far, legislation in relation to syphilis has been of but little benefit, on account of the impracticability of the laws passed.

Houses of prostitution are the most virulent centers, and the first blow must be struck at them. The total abolition of prostitution, though devoutly to be wished, is, at the present day, beyond the hope of man, and attempts which have been made towards accomplishing such an end present throughout centuries an uninterrupted series of defeats, for the sexual passion, the strongest impulse of human nature, has invariably asserted itself, and other laws have been violated ; seduction, criminal abortion, illegitimacy and infanticide, with impairment of morality, have followed the experiment.

Inspection of prostitutes by medical examiners has been enforced in many cities, and as often as it has been tried it has proven a failure ; for, as every one knows who possesses any knowledge of venereal diseases, it is folly to believe it possible to prevent the danger of infection by weekly examinations of the prostitutes of a city, as the inspector may find the woman free from disease on the day of inspection, only to have it appear during the interim ; furthermore, it is not always possible to detect the primary lesion of syphilis, and in consequence of these facts the system is rather more of a danger than a safeguard, for many young men, through a false sense of security, on learning that the prostitute possesses a certificate of health, will indulge in the sexual act when otherwise they would not.

Laws must be passed of so rigid a nature as to force the prostitute, through fear, to place a higher value on her health, and when the fight is once begun it must be persistently carried on with sufficient vigilance and energy to accomplish good results, by alarming the prostitute into viewing this condition in a more serious light. Strike at their freedom and you will gain their co-operation, for they will look upon syphilis as being something far more serious than a skin disease, and regard the probability of infecting others as worthy of consideration. Reckless of their health, devoid of any feeling for their fellow-being, they are jealous of their liberty, and, as do all criminal classes, entertain the profoundest respect and fear for the law.

The law must make a stern and relentless crusade against the syphilitic, striking first at the prostitute, and where found, forcing her to a place of confinement, where she can be kept under proper treatment until free from possibility of infecting others. It is a well-known fact that but few of these will take the proper treatment long enough to do much good, and it is very essential, to the accomplishment of our purpose, that some place be established to which syphilitics may be sent. We have the Isle of Molokai for the leper, why not find some such place for the syphilitic? The mere existence of such a place will have a greater moral effect than all the pulpit oratory and clinical teaching of the world combined. The search must extend beyond the bagnio, and find those men, "The Rounders," who are ever a source of infection for the prostitute; these also must be forced to take treatment which will relieve all danger of their speading the disease.

To be successful, the strong arm of the law must strike whereever syphilis exists, and should even enter the church, and strike at Hymen's altar, not permitting any man known to be syphilitic to embrace in connubial bliss the pure and healthy girl whom he wishes to make his wife. The marriage certificate should carry with it a clear bill of health, which should be the cardinal clause in its requirements.

It may be said by some that such interference with the liberty of the individual is illegal; yet we claim that the State has the right to protect the health of its citizens by every available means. Our quarantine laws against yellow fever, smallpox, etc., interfere with individual liberty, and the same should apply to syphilis, which, while not so dangerous to life, is still a terrible menace to health, and on account of it being, as it were, a concealed danger, with many modes of infection, it is greatly to be dreaded. Why should we hesitate to make open and relentless war upon these women, lost to all sense of morality, and so long permitted to practice their nefarious occupation, unmolested by the law, unnoticed by sanitarians, feeling no concern as regards the danger of their sexual companion, and who stand an awful menace to our young men and an indirect cause of untold suffering to the pure women of society, and the origin of a hereditary taint, which follows throughout generations, when we imprison the poor wretch who steals a loaf to keep off hunger? Is it a greater crime to pick a pocket than to steal your health, and that of your wife and your offspring?

The laity must be told the unvarnished truth, and warned of the many dangers of infection. The mock modesty of the law-maker and the unsound religion of the pulpit must be changed. Prostitution, though in a sense greatly to be deplored, is, in a way, absolutely essential; it should be under control of the law and kept clean as possible.—*Louisville Medical Journal.*

---

### TIME LIMIT.

Occasionally a letter is received in which the writer gravely says he is taking so many journals he cannot read the half of them, etc., the polite phrases being framed as an excuse for discontinuing his subscription, which is all right and in perfect accord with the privilege of every man who knows and attends to his own business, with which there is no occasion whatever for argument. It serves, however, as an introduction to the claim made that he has not time to read half the journals he is taking.

The time was, and is not so very far back, when the ordinary general practitioner of medicine thought he was doing entire justice to himself, his profession and his clients if he took a single medical journal. Conditions and times have greatly changed within the last twenty years. A medical education costs more than twice as much time and money as it did then, and the literature of medicine has quadrupled within that period. Friction and competition have increased in a similar ratio, so that the men who are ambitious to be at the front find themselves obliged to purchase new and improved instruments of precision, new books, and to take more than one or two medical journals. The man who has little business is always the one most crowded for time, and the little business he has is his boss.

The men who do the most work and accomplish great results always have time at their command, and always boss their business. They are rarely crowded, and seldom in a hurry. Visitors are received and made to feel a welcome that is not an intrusion, that time is easy, no fret or fidget; work is either going on or there is a timely rest. Such men take and read from ten to twenty or more journals. They don't pretend to read every article, but they carefully read those in which they are interested, and scan more or less closely the advertising pages. In the latter many useful hints are found; here they find reference to the tools of latest pattern and design, hence do not pretend to pass them by.

The known men in the profession are the very ones who can be most easily and successfully approached for any given purpose, whether it be to write an article or deliver a lecture, go on a journey or engage in a new enterprise. Their engagements are rarely pressing, and are always so dovetailed in time as to leave ample margins, and at the same time always fit in the right places.

The man who hasn't time to read half of the two journals he is taking is either fussing about a call he has to make or fretting because another doctor was called where he expected to officiate as chief factotum. In either instance his hours are so jaggered that he has little or no time to read, and that which he does read is not remembered.

Two things are never realized by the man who hasn't time; one is that there are even and exactly sixty minutes in every hour, and the other is like it, in which he fails to understand in its true bearings that there are neither more nor less than one hundred cents in every dollar.

The busiest men in the medical profession always attend the national, State and local medical societies. They are the ones who read papers, take part in the discussions, and then attend all the social functions. Not once are they disconcerted by pressing business engagements, a reason for which is found in the fact that just at that time affairs are so arranged that a pressure does not come.

The men who can't get off to attend such meetings are worried and perplexed for fear some rival will profit by their absence, instead of swapping time and business with such rivals, through which both would prosper and be better thought of. The world is wide, and there are other pebbles on the beach.

In joint stock corporations there are not infrequently two classes of stockholders—those who hold preferred and those who own common stock in the concern. The first class receive a stipulated dividend, not usually a large one, but it is assured out of the first earnings of the company. The common stock dividends are more precarious, and may be either smaller or larger than those paid to the preferred class. As a rule, preferred stock is preferred by investors—preferred because it is preferred and has less liability of loss.

Most current periodicals have two classes of subscribers—preferred and common. The preferred are preferred because they pay approximately in advance, are constituted of the men who have time to read and press their business, and do not allow their business to boss them. They go to association and society meetings, know there are sixty minutes in every hour and one hundred cents in every dollar. They know who and what is going on in the world, and are themselves right in the van. In the phraseology of the Alabama Congressman, they know where they are at. They know the publication they want, know the price of it and the advantage of prompt payment, not only to themselves, but to the publisher, and get lowest spot-cash rate.

The non-preferred or common subscriber, who is bossed by his business and hasn't time to read either books or journals, says to himself he will pay, if convenient, when a certain patron pays his bills, or when his ship comes from over the deep blue sea, and acts accordingly, so that dun follows dun. To meet time engagements and risk of absolute losses, where the patron has not paid nor the ship escaped foundering on unseen shoals, the subscription price is advanced, so that the delinquent has to pay interest for a withheld small sum.

The writer knows very well that there are unforeseen circumstances which make men do as they can and not as they desire, which obliges them to curtail on the necessaries of life as well as on luxuries which they crave, and with whom no fault can be found. They remind the writer of a recent conversation with a successful man in another profession, in which he said he believed men, most men, had a call—providential call—to some pursuit, by which he meant that Nature, more or less according to the loudness of the call, fitted them, and in which they generally achieved some success in life. Often a call was heard and accepted, whereas it was intended for another. There are men who enter the ministry erroneously, but perhaps honestly believing they are providentially called, when they heard only an echo that should have gone to another. He believed there were calls to enter the medical and other professions. In this opinion he was right. Every physician knows perfectly well that there are those in his profession who are not only unfitted by nature for their occupation, but are actually unhappy in it, and may generally be classed as failures. Fortunately, there is an elasticity in the well-rounded, well-balanced man, which enables him to surmount obstacles and to adapt himself to spheres of influence even in a life-work, where and when he would have wrought much more easily, and been more of a pronounced success, in another calling than the one which circumstances threw him into.—*Cincinnati Lancet-Clinic.*

---

[Reprinted from the Journal of the American Medical Association, July 1, 1899.]

### STANDARDIZATION NEEDED.

The revision of the "United States Pharmacopœia," which is to be done next year, is a matter of sufficient importance to receive the serious consideration of the profession. The physician is the one who has the greatest interest in the reliability and availability of the products of the pharmacist. They are part of the tools of his trade and on their efficiency his success to a greater or less extent depends. It is a well-known fact that many of the crude drugs that form the basis of the pharmacopœial preparations are far from being as reliable as their proper medical usage demands. When such physiologically powerful drugs, for example, as colchicum, conium, hydrastis, hyoscyamus, and others may vary in their content of active principle 200 to 300 per cent. in different samples, as has been amply demonstrated by competent authorities, it would seem that something ought to be done to eliminate these fluctuations of the crude drug from the official preparations. If the latter are not uniform in medical potency, what confidence can be placed in them or in the pharmacopœia, which certainly ought to be a reliable guide for accurate dosage and medication?

It is true, the last edition of the pharmacopœia did provide standards for cinchona, opium and nux vomica, and the recently published "British Pharmacopœia" goes a step further and "standardizes" ipecac and belladonna, but the principle has not yet been made to cover calabar, coca, colchicum, conium, gelseminum, hydrastis, podophyllum, stramonium or veratrum, to say nothing of important drugs such as aconite, cannabis indica, digitalis, ergot, and strophanthus, which defy any and every chemical test thus far elaborated, and which, to be assayed at all, must be tested pharmacologically on the living animal.

It is, perhaps, demanding too much to ask the revisers of the Pharmacopœia who are soon to enter on their decennial task, to provide standards for all powerful drugs, but it seems to be well within the bounds of the reasonable and moderate to urge the expediency of extending the principle of chemical standardization to all drugs susceptible of accurate chemical assay and also of adjustment, by chemical means, to uniform standards based on a fixed percentage of active principle or principles in the finished preparation. Surely, the physician has enough to perplex and baffle him in the idiosyncrasies of individual patients, and in the irremediable difficulties of diagnosis—may he not justly demand protection from the disaster which follows in the train of a weak, inert, unreliable drug product, or of a preparation possessing an unusual and dangerous potency? The golden mean between the worthless and toxic ought to characterize every fluid extract, solid extract or tincture administered in the treatment of disease.

The very general use of diphtheria antitoxin and the growing employment of an antitetanic serum for prophylactic purposes have acquainted the profession with the fact that the curative serums can be tested and standardized only by the physiologic method—by observing how much of the serum will preserve from sickness a test-animal into which is injected simultaneously ten times the fatal dose of the respective toxin. This is indeed a tedious, laborious, expensive, and not absolutely uniform means of pronouncing on the exact strength of a given serum, but it is the only means available; there is no other, as no chemist pretends that he can test a parcel of antitoxin with his reagents. Every word of this applies with almost equal force to the testing of a limited number of powerful and important drugs like ergot, digitalis, squill, convallaria majalis, cannabis indica and strophanthus. The chemical test for these drugs and their pharmaceutic preparations is very unreliable, and unless they are tested by the pharmacologist, on the living animal, their administration is a lottery affording no guarantee of prompt reaction or final cure. This fact is notoriously the cause of that unfortunate desuetude into which ergot and cannabis indica have largely fallen. Lacking uniformity of action and failing often to yield the expected results, the pharmaceutic preparations of the market are wholly discarded by the disappointed practitioner. Professor Hare, in his "Therapeutics," ascribes the frequent failure of cannabis indica to the inferiority of the preparations encountered, and the worthlessness of much of the ergot on the market is beyond dispute. Witness also the report of Houghton,* who pharmacologically tested six samples "supposed to be pure strophanthia;" one sample was *ninety* times as strong as another, and the remaining four varied between these limits of one and ninety.

If drug preparations can be made uniform in strength by the comparatively simple and inexpensive means of chemical assay, well and good, otherwise the physician has a right to ask the wealthy and prosperous manufacturer to apply the physiologic test to preparations whose aetivity can be gauged in no other manner.

* Journal, October 22, 1898.

